# The Prophylactic Protection of *P. acidilactici* M22 from Feline Milk on *S.* Typhimurium Infection in Mice

**DOI:** 10.3390/microorganisms13102353

**Published:** 2025-10-14

**Authors:** Xinyu Gong, Xue Wang, Lu Chen, Huiming Huang, Ning Zhang, Jun Han, Zhengping Wang, Min Wen

**Affiliations:** 1 Shandong Key Laboratory of Applied Technology for Protein and Peptide Drugs, Institute of Biopharmaceutical Research, Liaocheng University, Liaocheng 252000, China; pinkrose1998@163.com (X.G.); bioactiveschina@163.com (Z.W.); 2 Shandong Key Laboratory of Applied Technology for Protein and Peptide Drugs, School of Pharmaceutica Sciences and Food Engineering, Liaocheng University, 1 Hunan Street, Liaocheng 252059, China; hmhuang1988@163.com; 3 Pet Nutrition Research and Development Center, Gambol Pet Group Co., Ltd., Liaocheng 252000, China

**Keywords:** *P. acidilactici*, *S.* Typhimurium, gut microbiota, intestinal barrier

## Abstract

Breast milk is a major source of probiotics, particularly lactic acid bacteria (LAB), which are known to regulate the intestinal microbial community and exert antibacterial effects. However, little is known about the preventive effects of feline milk-derived LAB against Salmonella infection in vivo. In this study, a strain of *Pediococcus acidilactici* (M22) was isolated from feline milk and evaluated for its protective potential in C57BL/6 mice challenged with *Salmonella* Typhimurium SL1344 (VNP20009). Following oral administration of M22, mice were infected with *S.* Typhimurium, and protective efficacy was assessed through body weight changes, bacterial loads in tissues, histopathological examination of the colon, oxidative stress markers, cytokine profiles, and 16S rRNA gene sequencing of cecal microbiota. The results showed that pretreatment with M22 significantly reduced bacterial loads in the liver, spleen, and cecum compared with controls. M22 administration enhanced antioxidant capacity, alleviated infection-induced inflammation, and preserved intestinal barrier integrity by restoring villus morphology and upregulating tight junction proteins (ZO-1 and occludin). Microbiota analysis further revealed that M22 enriched short-chain fatty acid-producing beneficial taxa (e.g., lactic acid bacteria) while suppressing pro-inflammatory genera. Collectively, these findings provide scientific evidence that feline milk-derived *P. acidilactici* M22 is a safe and effective probiotic candidate. By enhancing gut health and host resistance to infection, M22 offers a promising strategy to improve companion animal health, reduce reliance on antibiotics, and mitigate zoonotic transmission of pathogens.

## 1. Introduction

In recent years, with changes in lifestyle and emotional needs, the companionship role of domestic cats (*Felis catus*) has become increasingly prominent, and their global population has expanded substantially [1]. As a result, feline health management has become a major focus in both veterinary medicine and public health [2]. Early-life nutrition is now recognized as a key determinant of long-term health, as this period represents a critical developmental window during which the gastrointestinal tract, immune system, and gut microbiota remain immature and highly susceptible to external influences [3]. Gastrointestinal disorders, particularly those caused by enteric pathogens such as *Salmonella* Typhimurium, are still common in young *cats* [4]. Early-life gastrointestinal infections not only impair growth and immune function but also disrupt the establishment of a balanced gut microbiota, leading to long-term adverse health outcomes [5]. Currently, antibiotics remain the primary strategy for treating bacterial enteric infections [6,7,8]. However, the administration of antibiotics during early life has been shown to cause multiple long-term negative effects, including disruption of gut microbiota composition, increased risk of metabolic disorders, impaired immune development, and the emergence of antibiotic-resistant bacteria [9]. These limitations underscore the urgent need for alternative strategies that can safely and effectively modulate the gut microbiota and enhance host defenses during this critical developmental stage.

Breast milk is widely regarded as the optimal source of nutrition for newborns, providing not only balanced macro- and micronutrients essential for growth but also a repertoire of bioactive compounds and beneficial microorganisms [10]. Antimicrobial components in milk, such as lysozyme and lactoferrin, have been demonstrated to inhibit Salmonella, thereby contributing to the protection of suckling kittens [11]. However, during weaning, kittens lose these protective antimicrobial factors derived from milk and simultaneously undergo a dietary transition from milk to solid food. This transition frequently leads to physiological gastrointestinal disturbances, reflecting the incomplete development of the gut and immune system. Milk from both humans and animals, including feline milk, has been shown to harbor diverse bacterial communities, particularly lactic acid bacteria (LAB) such as *Lactobacillaceae* [12], *Bifidobacterium* [13]. These milk-derived microbes can colonize the neonatal gut, inhibit the adhesion and proliferation of pathogens, and thus play a pivotal role in protection against early-life infections [14]. Recent evidence indicates that milk-derived components, especially milk fat globule membranes (MFGM), can substantially enhance the adhesion and survival of probiotic bacteria within the intestinal epithelium. For example, Wasana et al. (2025) demonstrated that the presence of MFGM increases the expression of adhesion-related genes (e.g., *MapA*, *Slp*) and thereby reduces pathogen colonization on the gut lining [15]. These features make them highly attractive candidates for functional food development or therapeutic applications, particularly in newborns and young animals with immature intestinal ecosystems that are more vulnerable to pathogenic invasion.

*S.* Typhimurium is a widespread foodborne pathogen that poses a significant threat to both human and animal health. Several LAB species, including Lacticaseibacillus rhamnosus, Lactiplantibacillus plantarum [12], and Bifidobacterium [16], have been shown to inhibit *S.* Typhimurium through mechanisms such as competition for adhesion sites, secretion of antimicrobial metabolites, and modulation of host immune responses. Among them, *P. acidilactici* has recently attracted growing attention owing to its strong tolerance to acid and bile salts, high adhesion capacity to intestinal epithelial cells, and ability to produce antimicrobial metabolites such as lactic acid and bacteriocins [17]. In addition, *P. acidilactici* contributes to immune regulation, mitigation of oxidative stress, and maintenance of intestinal barrier integrity, making it a promising probiotic candidate for disease prevention and gut health promotion [18]. Nevertheless, reports on *P. acidilactici* strains derived from feline milk remain scarce. This knowledge gap highlights the necessity of exploring whether milk-derived LAB can serve as a safe and effective probiotic strategy to prevent early-life enteric infections in *cats*.

In this study, we investigated the preventive effect of *P. acidilactici* M22, a strain isolated from feline milk, on *S.* Typhimurium SL1344-induced intestinal infection in C57BL/6 mice. We assessed its impact on clinical outcomes, intestinal histopathology, inflammatory cytokines, oxidative stress biomarkers, and tight junction proteins. This research aims to provide experimental evidence supporting the application of *P. acidilactici* M22 as a potential probiotic candidate for preventing Salmonella infections, thereby reducing antibiotic dependence and addressing the growing concern of zoonotic transmission in pet–human ecosystems.

## 2. Materials and Methods

### 2.1. Source and Preparation of Bacterial Strains

*S.* Typhimurium SL1344 was purchased from BIO SCI Technology Co., Ltd. (Qingdao, China). *P. acidilactici* M22 (CCTCC NO. 31001), previously isolated and identified by our laboratory, was utilized in this study. Bacterial cultures were grown in MRS broth at 37 °C and harvested during the logarithmic growth phase [17]. The optical density at 600 nm (OD600) was measured, and cell suspensions were normalized to a concentration of 1 × 10^9^ CFU/mL based on a pre-established OD600–CFU correlation curve before subsequent experimental procedures. The probiotic strain *P. acidilactici* M22 was cultured overnight in MRS broth (Haibo, China) at 37 °C. *S.* Typhimurium SL1344 was cultured in Luria–Bertani (LB) broth at 37 °C with shaking at 120 rpm overnight [19].

### 2.2. Experimental Design

Thirty 6-week-old male C57BL/6 mice were housed under standard laboratory conditions (temperature 22–26 °C, humidity ~20%, and a 12 h light/dark cycle) with ad libitum access to standard feed and distilled water. After one week of acclimatization, the mice were randomly divided into five groups (*n* = 6): a control group (C), a Salmonella infection model group (MG), and three probiotic treatment groups designated as low (L), medium (M), and high (H) [20]. During the first 14 days, mice in the probiotic groups (L, M, and H) were orally administered 200 µL of *P. acidilactici* M22 suspension at concentrations of 1 × 10^7^, 1 × 10^8^, and 1 × 10^9^ CFU/mL, respectively. An equal volume of sterile phosphate-buffered saline (PBS) was administered to the C and MG groups. On day 15, all groups except the C group were challenged orally with 200 µL of *S.* Typhimurium *SL1344* suspension (5 × 10^8^ CFU/mL). After infection, mice were observed daily for five consecutive days, and clinical indicators including body weight, blood in stool, mental status, and physical activity were recorded. Upon completion of the study, all mice were humanely euthanized in accordance with ethical guidelines to facilitate tissue collection and downstream analysis.

### 2.3. Monitoring of Body Weight Loss

Body weight of each mouse was recorded daily post-infection to monitor health status. Body weight loss curves were plotted and analyzed to evaluate disease severity and recovery [21].

### 2.4. Determination of Salmonella Translocation

The liver, spleen, and cecum from each mouse were aseptically collected, weighed, and homogenized in sterile PBS (1:10, *w*/*v*). The homogenates were serially diluted, and appropriate dilutions were plated onto bismuth sulfite agar medium (Haibo, Qingdao, China). Plates were incubated at 37 °C for 24 h for enumeration of Salmonella-specific colonies. The threshold for bacterial translocation is expressed as log10 CFU/g of each organ sample [21].

### 2.5. Measurement of Serum Inflammatory Cytokines

Blood samples were collected by cardiac puncture and centrifuged at 5000 rpm for 15 min at 4 °C to obtain serum. Serum levels of key inflammatory cytokines, including TNF-α, IL-1β, IL-6, and IL-10, were measured using commercially available enzyme-linked immunosorbent assay (ELISA) kits in strict accordance with the manufacturer’s protocols [22].

### 2.6. Assessment of Oxidative Stress Markers in Jejunum

Jejunum segments were harvested, homogenized in ice-cold PBS, and centrifuged at 12,000 rpm for 10 min at 4 °C. Supernatants were collected for the assessment of oxidative stress markers—malondialdehyde (MDA), superoxide dismutase (SOD), and glutathione (GSH)—using commercial assay kits in accordance with the manufacturer’s protocols [23].

### 2.7. Histological Analysis

Jejunum tissue samples were immersed in 10% neutral-buffered formalin for fixation, subsequently embedded in paraffin, and sliced into 5 μm sections. These sections were then subjected to hematoxylin and eosin (H&E) staining to facilitate histopathological evaluation. Tissue sections were evaluated under a light microscope for morphological changes.

### 2.8. Immunofluorescence Staining for Intestinal Tight Junction Proteins

Jejunum tissue sections were deparaffinized, subjected to antigen retrieval, and blocked with 5% bovine serum albumin (BSA). Sections were then incubated overnight at 4 °C with primary antibodies against Occludin and ZO-1. After washing, sections were incubated with fluorescence-labeled secondary antibodies, counterstained with DAPI for nuclear visualization, and observed using confocal microscopy.

### 2.9. Analysis of Gut Microbiota

#### 2.9.1. DNA Extraction and PCR Amplification

Total microbial genomic DNA was isolated from the samples with the E.Z.N.A.^®^ soil DNA Kit (Omega Bio-tek, Norcross, GA, USA) following the manufacturer’s protocol [24]. DNA purity and concentration were assessed by 1.0% agarose gel electrophoresis and a NanoDrop2000 spectrophotometer (Thermo Scientific, Waltham, MA, USA), and samples were stored at −80 °C until further analysis [25]. The V3–V4 hypervariable region of the bacterial 16S rRNA gene was amplified employing primer pairs 338F (5′-ACTCCTACGGGAGGCAGCAG-3′) and 806R (5′-GGACTACHVGGGTWTCTAAT-3′) [1] using a T100 Thermal Cycler (BIO-RAD, Hercules, CA, USA). Standard PCR amplification was performed using specific primers and a commercial master mix. The resulting products were then visualized via electrophoresis, purified, and quantified fluorometrically for downstream applications [26].

#### 2.9.2. Data Processing

Raw FASTQ data underwent demultiplexing, quality filtering, and assembly using custom scripts and bioinformatics tools. Quality-controlled sequences were then clustered into OTUs at 97% similarity, followed by removal of chloroplast-derived sequences. To mitigate sequencing depth bias, all samples were rarefied to 20,000 sequences per sample [26].

Taxonomic assignment of OTUs was performed using the RDP Classifier (v11.5) against the Silva v138.2 database with a confidence threshold of 0.7. Metagenomic functions were predicted with PICRUSt2 (https://cloud.majorbio.com, accessed on 25 May 2025), which placed representative sequences into a reference tree using EPA-NG (https://cloud.majorbio.com, accessed on 25 May 2025) and Gappa (https://cloud.majorbio.com, accessed on 25 May 2025), normalized copy numbers with Castor (https://cloud.majorbio.com, accessed on 25 May 2025), and predicted gene pathways via MinPath (https://cloud.majorbio.com, accessed on 25 May 2025), following the recommended workflow [27,28,29,30].

### 2.10. Statistical Analysis

Data are expressed as mean ± SEM. All statistical analyses were performed with GraphPad Prism 9.0 (GraphPad Software, San Diego, CA, USA). An unpaired *t*-test was applied for comparisons between two groups, whereas one-way ANOVA was used for comparisons among multiple groups. Differences were considered statistically significant at *p* < 0.05.

## 3. Results

### 3.1. P. acidilactici M22 Pretreatment Attenuated Body Weight Loss Induced by S. Typhimurium

**Figure 1:**
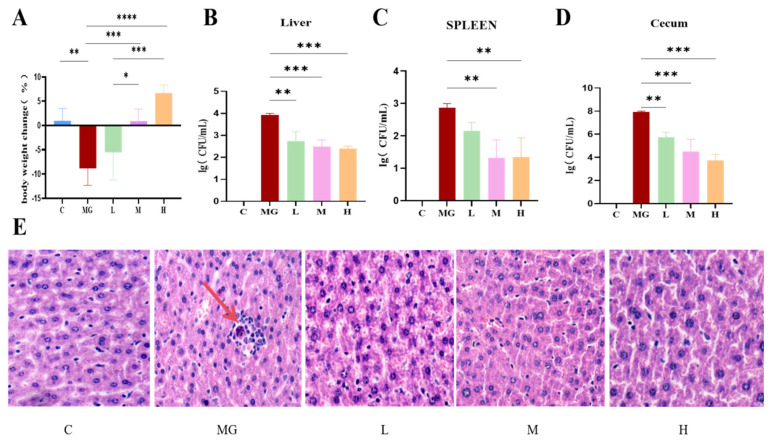
Effect of probiotic pretreatment on body weight, bacterial load in tissues, and liver histopathology in mice infected with *S.* Typhimurium. (**A**) Body weight change (%) in each group: control; (**B**–**D**) Bacterial load (log CFU/mL) in the liver (**B**), spleen (**C**), and cecum (**D**) of mice from different groups. (**E**) Representative H&E-stained liver sections from each group (magnification ×20). C: normal hepatic architecture with well-arranged hepatocyte cords; MG: obvious hepatocellular swelling, inflammatory cell infiltration, and disordered hepatic cords; L-H: probiotic pretreatment alleviated histopathological damage in a dose-dependent manner. Data are expressed as mean ± SEM, and statistical differences are indicated as * *p* < 0.05, ** *p* < 0.01, *** *p* < 0.001, and **** *p* < 0.0001.

Mice in the MG group exhibited a marked reduction in body weight compared to the C group after *S.* Typhimurium infection (*p* < 0.01). In contrast, probiotic pretreatment significantly mitigated this weight loss, with the H group demonstrating the most pronounced effect (*p* < 0.05) (Figure 1A).

### 3.2. The Probiotic P. acidilactici M22 Reduces Translocation of S. Typhimurium

Bacterial load analysis revealed that the MG group had significantly higher colony counts in the liver (*p* < 0.001), spleen (*p* < 0.01), and cecum (*p* < 0.001) compared with the C group. Probiotic pretreatment significantly decreased bacterial loads in all examined tissues, and this reduction exhibited a dose-dependent trend (Figure 1B–D).

### 3.3. P. acidilactici M22 Attenuates S. Typhimurium–Induced Hepatic Histopathological Damage

Representative H&E-stained liver sections showed distinct histopathological differences among groups (Figure 1E). The C group exhibited normal hepatic lobule architecture, with hepatocytes arranged radially around the central vein and no inflammatory infiltration. In contrast, the MG group displayed severe hepatic injury, characterized by hepatocellular swelling, vacuolar degeneration, disordered hepatic cords, and extensive inflammatory cell infiltration (indicated by red arrows). Pretreatment with *P. acidilactici* M22 alleviated these pathological changes in a dose-dependent manner. The L group showed mild inflammatory infiltration and partial preservation of hepatocyte morphology, while the M group demonstrated further improvement, with reduced hepatocellular swelling and more orderly hepatic cords. The H group exhibited near-normal histology, with minimal inflammatory changes and largely restored hepatic architecture.

### 3.4. P. acidilactici M22 Attenuates S. Typhimurium–Induced Inflammation and Oxidative Stress

**Figure 2:**
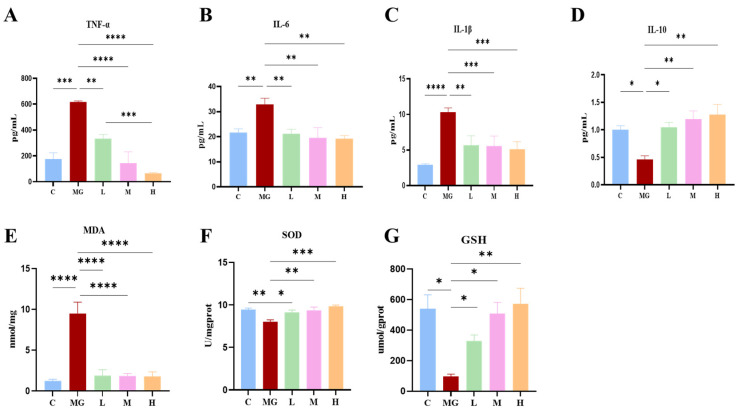
Effects of probiotic pretreatment on inflammatory cytokines and oxidative stress markers in *S.* Typhimurium-infected mice. Serum levels of pro-inflammatory cytokines (**A**) TNF-α, (**B**) IL-6, and (**C**) IL-1β, and the anti-inflammatory cytokine (**D**) IL-10. Oxidative stress indicators in liver tissue: (**E**) malondialdehyde (MDA), (**F**) superoxide dismutase (SOD), and (**G**) glutathione (GSH,). Data are expressed as mean ± SEM. Statistical significance is indicated as * *p* < 0.05, ** *p* < 0.01, *** *p* < 0.001, and **** *p* < 0.0001.

The MG group exhibited significantly elevated levels of TNF-α, IL-6, and IL-1β compared with the C group (*p* < 0.01 or *p* < 0.001) (Figure 2). Conversely, IL-10 levels were considerably reduced in the MG group (*p* < 0.05). Pretreatment with *P. acidilactici* M22 effectively mitigated these inflammatory changes in a dose-dependent manner. Specifically, the M and H groups demonstrated a significant reduction in TNF-α, IL-6, and IL-1β levels compared with the MG group, while IL-10 concentrations were restored toward normal levels (*p* < 0.01).

Similarly, the MG group exhibited markedly elevated MDA levels and significantly reduced antioxidant enzymes (SOD and GSH) compared with the control group (*p* < 0.001 or *p* < 0.0001). In contrast, *P. acidilactici* M22 pretreatment significantly attenuated oxidative stress, as evidenced by reduced MDA levels (*p* < 0.0001) and enhanced SOD and GSH activities, particularly in the M and H groups.

Collectively, these data illustrate that prophylactic administration of *P. acidilactici* M22 effectively alleviates inflammatory responses and oxidative stress induced by *S.* Typhimurium infection, supporting its therapeutic potential in mitigating pathogen-induced intestinal damage.

### 3.5. Effects of P. acidilactici M22 on the Intestinal Barrier

**Figure 3:**
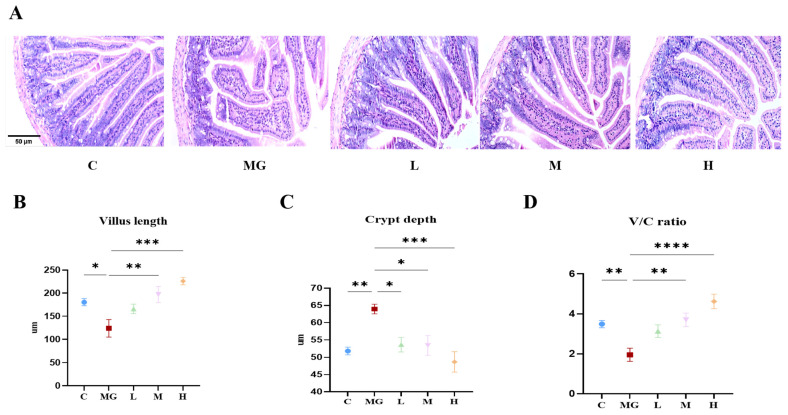
Effects of *P. acidilactici* M22 pretreatment on jejunal morphology and tight junction protein expression in *S.* Typhimurium–infected mice. (**A**) Representative H&E-stained jejunal sections from C, MG, and probiotic-pretreated groups at L, M, and H doses. (**B**) Quantification of villus length. (**C**) Quantification of crypt depth. (**D**) Villus-to-crypt (V/C) ratio. Data are expressed as mean ± SEM (*n* = 6). Statistical significance is indicated as * *p* < 0.05, ** *p* < 0.01, *** *p* < 0.001, and **** *p* < 0.0001.

As shown in Figure 3A, H&E staining revealed that jejunal villi in the C group were intact, were regularly arranged, and displayed moderate crypt depth. In contrast, the MG group exhibited obvious villus atrophy and disorganization accompanied by deepened crypts, indicating severe intestinal injury. Administration of *P. acidilactici* M22 at low (L), medium (M), and high (H) doses markedly improved the jejunal morphology compared with the MG group, as reflected by elongated villi and reduced crypt depth, with the effect of greatest magnitude observed in the H group.

Quantitative analysis further validated these observations (Figure 3B–D). Villus height was significantly reduced in the model group, whereas crypt depth was markedly increased, leading to a sharp decline in the villus-to-crypt (V/C) ratio. In contrast, supplementation with *P. acidilactici* M22 significantly increased villus height and decreased crypt depth, thereby elevating the V/C ratio. These results indicate that *P. acidilactici* M22 effectively alleviates *S.* Typhimurium-induced jejunal damage and restores intestinal absorptive function and barrier architecture.

**Figure 4:**
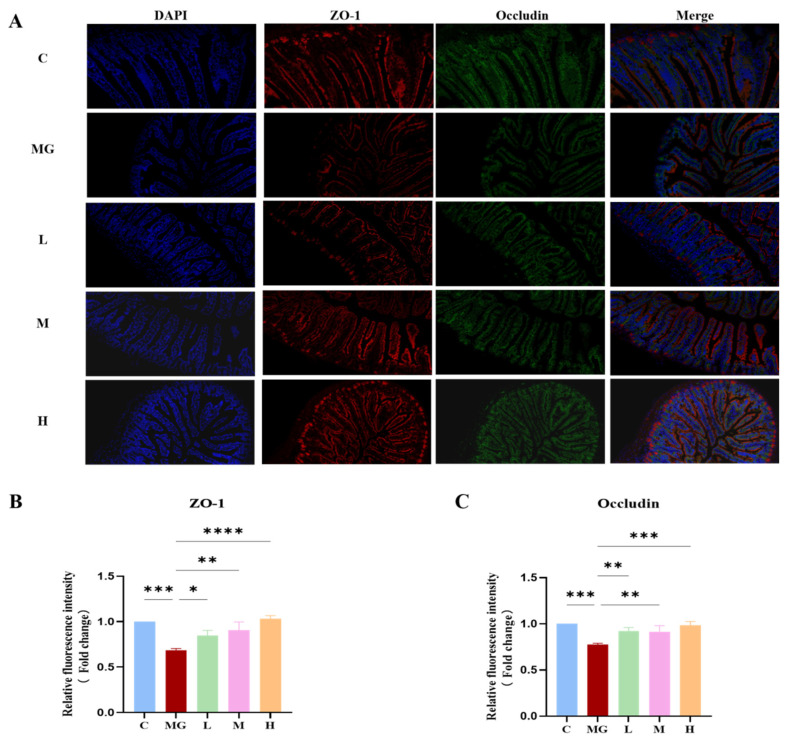
Effects of M22 pretreatment on the expression of intestinal tight junction proteins in *S.* Typhimurium-infected mice. (**A**) Representative immunofluorescence staining of Jejunum sections. Nuclei were stained with DAPI (blue), ZO-1 was labeled with red fluorescence, and Occludin was labeled with green fluorescence. Merged images show the colocalization of nuclei and tight junction proteins. (**B**) Relative fluorescence intensity of ZO-1 and (**C**) Occludin. Statistical significance is indicated as * *p* < 0.05, ** *p* < 0.01, *** *p* < 0.001, and **** *p* < 0.0001.

To investigate the role of *P. acidilactici* M22 in preserving intestinal barrier integrity, immunofluorescence staining for ZO-1 (red) and Occludin (green) in the jejunum was performed (Figure 4A). Immunofluorescence analysis revealed that *S.* Typhimurium infection markedly reduced the expression of tight junction proteins ZO-1(Figure 4B) and Occludin (Figure 4C) in the jejunal epithelium compared with the control group (*p* < 0.01). In contrast, pretreatment with *P. acidilactici* M22 significantly restored the fluorescence intensity of both proteins in a dose-dependent manner. Notably, mice in the high-dose group exhibited fluorescence levels comparable to or exceeding those of the control group, indicating that M22 effectively preserved intestinal barrier integrity during pathogenic challenge. The C group exhibited intact and continuous expression of ZO-1 and Occludin along the intestinal epithelium. In contrast, the Salmonella infection MG group demonstrated severely disrupted localization and significantly reduced fluorescence intensity of these tight junction proteins, indicative of compromised barrier integrity. Pretreatment with *P. acidilactici* M22 significantly enhanced the expression of ZO-1 and Occludin in a manner correlated with dosage. Specifically, the L group showed partial recovery with increased fluorescence intensity and more continuous distribution compared with MG. The M and H groups exhibited notably improved expression patterns, characterized by strong, linear, and continuous staining along the intestinal epithelial layer, closely resembling the C group. These results suggest that *P. acidilactici* M22 pretreatment effectively restores intestinal tight junction integrity, thereby providing significant protection against *S.* Typhimurium-induced intestinal barrier dysfunction.

### 3.6. P. acidilactici M22 Alters Intestinal Microbiota Composition in Mice

#### 3.6.1. Alpha Diversity and Beta Diversity

**Figure 5:**
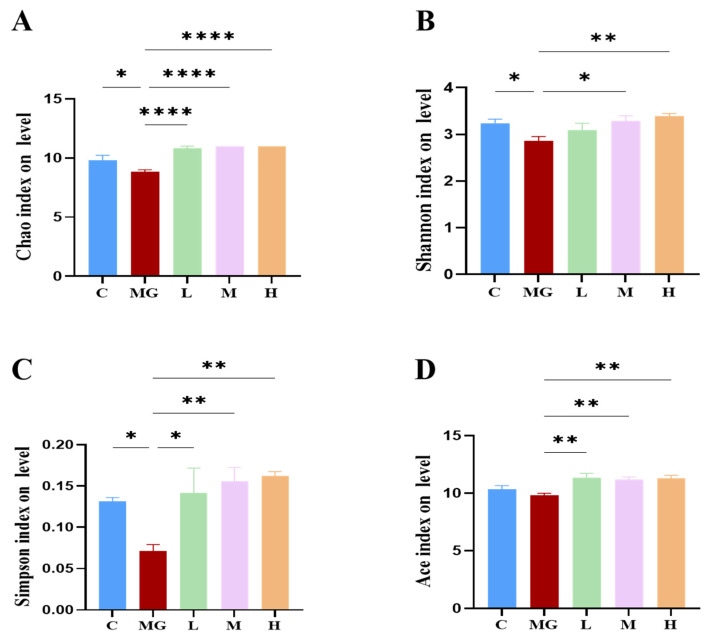
Effects of probiotic pretreatment on alpha diversity of gut microbiota in *S.* Typhimurium-infected mice. Alpha diversity indices, including (**A**) Chao1, (**B**) Shannon, (**C**) Simpson, and (**D**) Ace, were calculated to assess microbial richness. Data are presented as mean ± SEM, with significance levels indicated as * *p* < 0.05, ** *p* < 0.01 and **** *p* < 0.0001.

To assess the effects of probiotic pretreatment on the gut microbial community, alpha diversity indices (Chao1, Shannon, Simpson, and Ace) were analyzed (Figure 5A–D). *S.* Typhimurium infection significantly reduced the Chao1 index (*p* < 0.05) compared with the C group, indicating a loss of microbial richness. All probiotic pretreatment groups (L, M, H) exhibited significantly higher Chao1 values than the MG group (*p* < 0.0001), approaching control levels.

**Figure 6:**
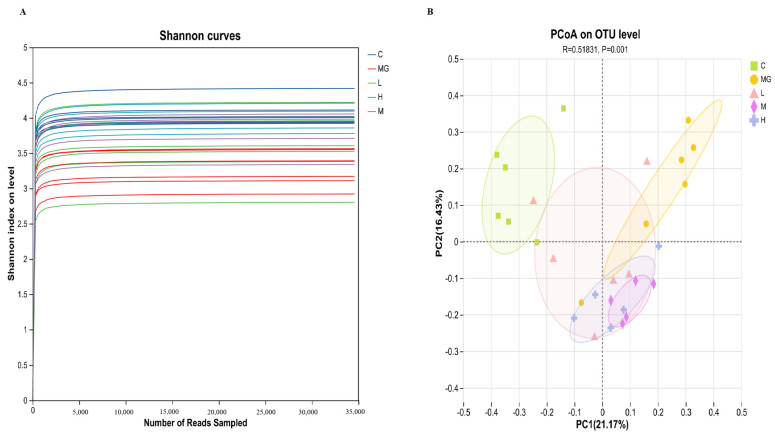
Effects of probiotic pretreatment on beta diversity of gut microbiota in *S.* Typhimurium-infected mice. (**A**) Shannon rarefaction curves showing species diversity saturation for all groups. (**B**) Principal coordinates analysis (PCoA) based on OTU level illustrating distinct clustering among groups. The MG group formed a separate cluster from the C and probiotic-treated groups, while probiotic pretreatment shifted microbial community composition toward that of the healthy C group. Statistical analysis confirmed significant separation among groups (R = 0.51831, *p* = 0.001).

Similarly, the Shannon index was significantly decreased in MG (*p* < 0.05), reflecting reduced diversity and evenness. Probiotic pretreatment increased the Shannon index, with the H group showing a significant improvement compared with MG (*p* < 0.01). A significant reduction in the Simpson index was observed in the MG group compared to the C group (*p* < 0.05), indicating a reduction in community diversity and evenness following *S.* Typhimurium infection. Probiotic pretreatment markedly increased the Simpson index in a dose-dependent manner, with all probiotic groups (L, M, H) showing significantly higher values than MG (*p* < 0.01) and approaching or exceeding control levels. These results indicate that probiotic treatment successfully reversed the infection-induced disruption of gut microbial diversity and evenness. The Ace index showed a similar trend to Chao1, with MG displaying a significant reduction (*p* < 0.01) that was reversed by probiotic administration (*p* < 0.01). Shannon rarefaction curves (Figure 6A) reached a plateau for all groups, confirming sufficient sequencing depth.

Beta diversity analysis was performed to evaluate differences in microbial community composition among groups. Principal coordinates analysis (PCoA) based on OTU level (Figure 6B) revealed distinct clustering of the MG group, clearly separated from the C and probiotic-treated groups. Notably, the microbial profiles of probiotic-pretreated mice were shifted toward those of healthy controls, with the most pronounced overlap observed in the high-dose group. This separation was statistically supported (R = 0.51831, *p* = 0.001), indicating that probiotic pretreatment effectively altered the gut microbiota composition disrupted by *S.* Typhimurium infection.

Collectively, these results demonstrate that *S.* Typhimurium infection markedly impaired gut microbial richness, diversity, and community structure, while probiotic pretreatment effectively restored alpha diversity and modulated beta diversity in a dose-dependent manner toward a healthier microbial profile.

#### 3.6.2. Cecal Microbiota Composition Following *S.* Typhimurium Infection

**Figure 7:**
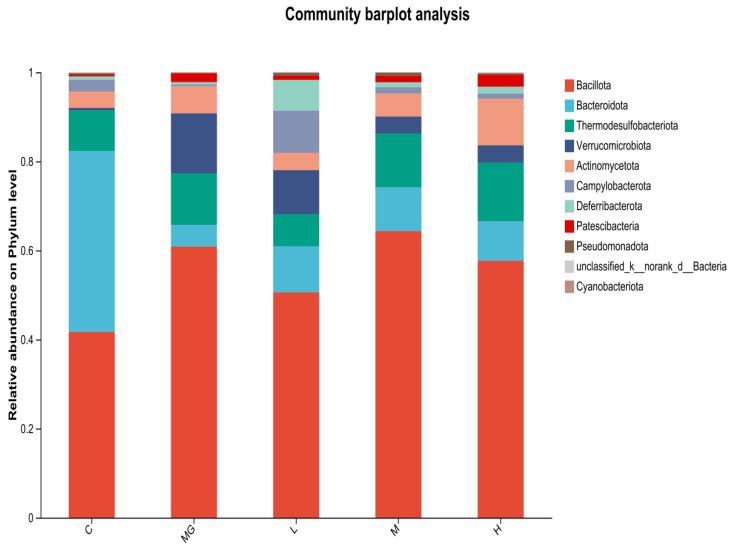
Relative abundance of cecal microbiota at the phylum level in *S.* Typhimurium-infected mice with or without probiotic pretreatment.

At the phylum level (Figure 7), 16S rRNA gene sequencing revealed that Bacillota and Bacteroidota were the dominant taxa in all groups. In the C group, the proportion of Bacteroidota was relatively high, whereas *S.* Typhimurium infection (MG group) resulted in a marked increase in *Bacillota* and *Verrucomicrobiota*, accompanied by a decrease in *Bacteroidota*. Probiotic pretreatment (L, M, H groups) partially restored the relative abundance of *Bacteroidota* and reduced *Verrucomicrobiota* compared with the MG group. These three phyla—Bacillota, Bacteroidota, and Verrucomicrobiota—represent major components of the normal intestinal microbiota.

**Figure 8:**
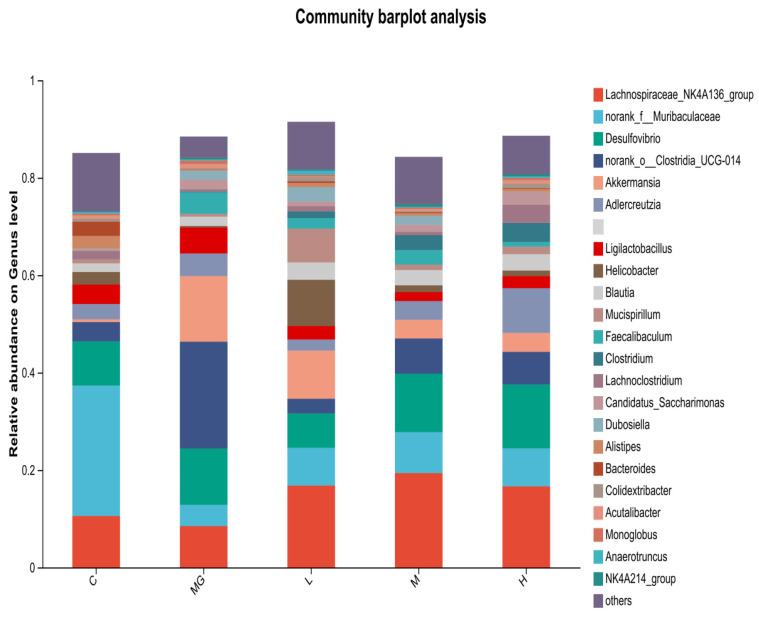
Relative abundance of cecal microbiota at the genus level in *S.* Typhimurium-infected mice with or without probiotic pretreatment.

At the genus level (Figure 8), Muribaculaceae and Lachnospiraceae were abundant in the C group. The MG group exhibited a reduction in Muribaculaceae and an increase in Akkermansia and Clostridia compared with controls. Probiotic pretreatment increased the relative abundance of beneficial genera such as Lachnospiraceae, Muribaculaceae, and Ligilactobacillus, while decreasing potentially harmful genera including Desulfovibrio and Helicobacter. Notably, Akkermansia abundance was higher in the MG group than in the probiotic-treated groups, whereas Ligilactobacillus was more abundant in the L, M, and H groups than in MG.

**Figure 9:**
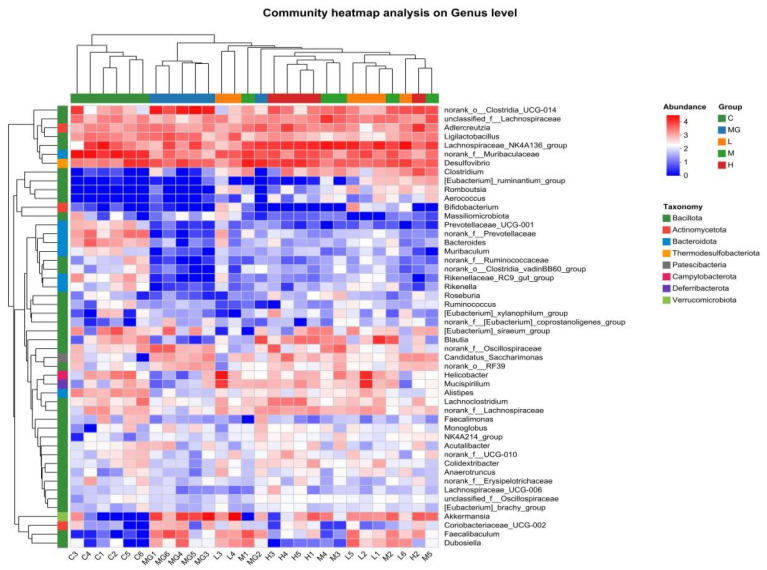
Heatmap plot depicting the relative abundance of each bacterial genus. In the figure, the samples were clustered by UPGMA according to the Euclidean distance of species composition data.

To compare microbial composition across groups, a genus-level heat map was constructed. Analysis revealed a gradation in the abundance of *Lachnospiraceae NK4A136* group, which was highest in the H group, intermediate in the L and M groups, and lowest in the MG group. Similarly, higher relative abundances of *Ligilactobacillus* and *Muribaculaceae* were observed in the L, M, and H groups compared to the MG group. Conversely, the MG group exhibited greater abundances of *Akkermansia*, *Helicobacter*, and *Desulfovibrio* relative to the probiotic-treated groups. Notably, the relative abundance of *Dubosiella* was highest in the MG group, significantly surpassing that in the L, M, and H groups (Figure 9).

#### 3.6.3. LEfSe Analysis

**Figure 10:**
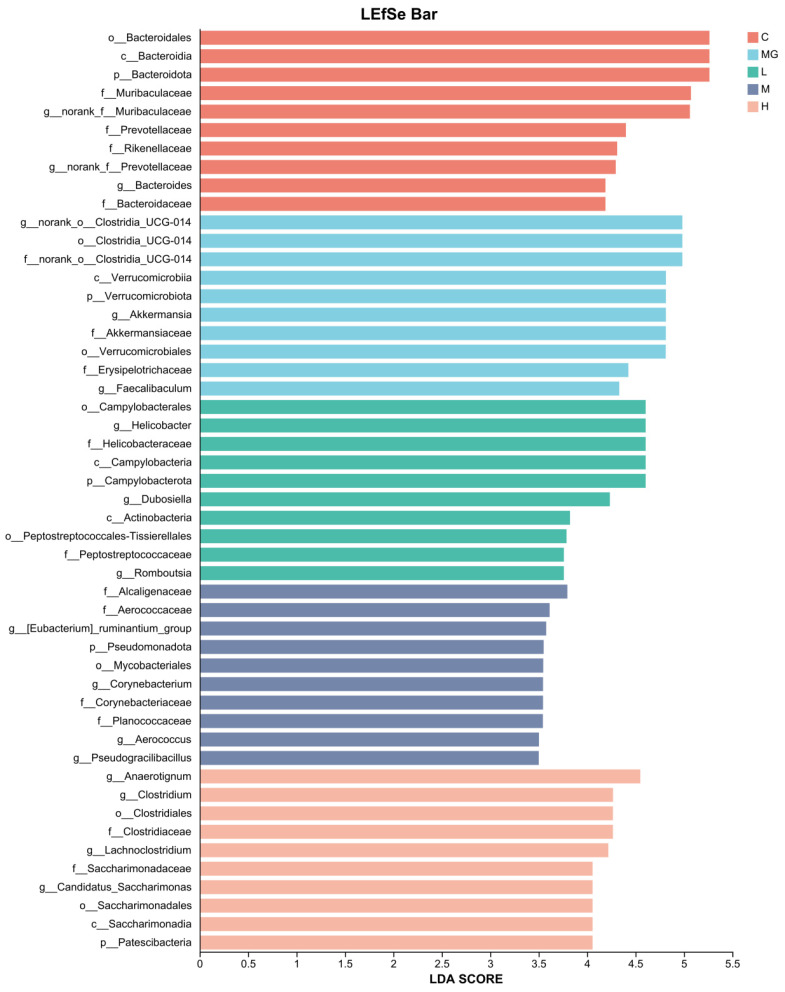
LDA scores obtained from the LEfSe analysis of the gut microbiota in different groups.

To investigate the differences in key bacterial taxa among groups, we performed an LEfSe analysis. The LDA score obtained from LEfSe analysis confirmed and visualized the characteristic microbial biomarkers of each group (Figure 10). In the C group, taxa such as *Bacteroidales*, *Bacteroides*, and members of *Muribaculaceae* and *Prevotellaceae* were significantly enriched compared with other groups. The MG group was characterized by a higher abundance of *Clostridia UCG-014*, *Verrucomicrobiota*, *Akkermansiaceae*, and *Akkermansia*, including *A. muciniphila*.

In the L group, enriched taxa included *Campylobacterales*, *Helicobacteraceae*, and *Helicobacter*. The H group showed higher abundances of *Dubosiella*, *Actinobacterota*, *Peptostreptococcales-Tissierellales*, and *Clostridium*. The M group was enriched in *Patescibacteria*, *Eubacterium_ruminantium_group*, *Aerococcaceae*, *Corynebacteriaceae*, and *Corynebacterium*. These results suggest that *S.* Typhimurium infection and probiotic pretreatment distinctly alter the gut microbiota composition, with specific taxa serving as potential biomarkers for each treatment group.

#### 3.6.4. Predicted Functional Profiling of the Gut Microbiome by PICRUSt2

**Figure 11:**
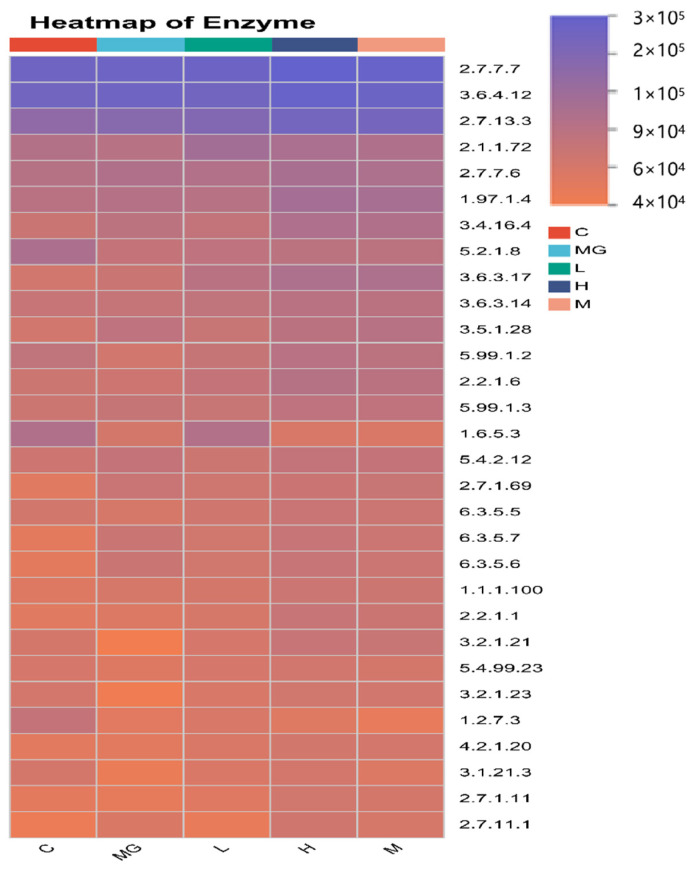
Heatmap of Predicted KEGG Enzyme Abundance Based on PICRUSt2 Analysis.

To characterize the functional capacity of the gut microbiota, PICRUSt2 analysis was performed to forecast KEGG pathway, and a heatmap was generated to display the relative abundance of functional enzyme categories across groups (Figure 11). *S.* Typhimurium infection (MG group) altered the abundance of multiple predicted microbial enzymes compared with the C group, particularly those involved in carbohydrate metabolism (e.g., EC 2.7.7.7, EC 3.6.4.12), amino acid metabolism (e.g., EC 2.1.1.72, EC 1.97.1.4), and energy production pathways (e.g., EC 5.1.2.8, EC 1.1.1.100).

These findings indicate that *S.* Typhimurium infection disrupts key microbial metabolic functions, while probiotic intervention can partially or fully restore the functional potential of the gut microbiome, with a dose-dependent effect.

## 4. Discussion

*S.* Typhimurium infection poses a significant public health challenge worldwide, particularly due to the zoonotic potential from companion animals such as cats and dogs [31]. Given the increasing number of companion animals and the associated risk of zoonotic pathogen transmission, there is a growing imperative to seek out viable alternatives to traditional antibiotic treatments that are both safe and effective, which are increasingly compromised by the rise of antibiotic-resistant bacterial strains [32]. Probiotics, especially lactic acid bacteria (LAB), have garnered attention for their ability to inhibit pathogens, modulate immune responses, and reinforce intestinal barrier integrity [33]. In our previous experiments, we observed that *P. acidilactici* M22 exhibited a pronounced inhibitory effect against *S.* Typhimurium SL1344 under in vitro conditions [17]. Our study specifically evaluated *P. acidilactici* M22, a LAB strain isolated from feline milk, demonstrating its preventive efficacy against *S.* Typhimurium infection in C57BL/6 mice.

Consistent with previous findings, we observed that prophylactic administration of *P. acidilactici* M22 effectively mitigated the clinical symptoms associated with Salmonella infection, including body weight loss and deteriorated general condition. These results align with prior research, which reported probiotics’ capability to prevent weight loss and enhance overall health status following pathogenic challenges [34]. For instance, Wu et al. demonstrated similar protective effects with Lactobacillus strains against Salmonella infections, highlighting the strain-specific efficacy of probiotics [35]. Our findings thus reinforce the importance of selecting host-derived probiotic strains, such as M22, to achieve optimal protective effects against pathogen-induced weight loss and clinical deterioration.

A critical aspect of Salmonella pathogenicity involves translocation from the gut to systemic organs, resulting in hepatosplenomegaly and systemic infections [36]. In our study, administration of *P. acidilactici* M22 markedly reduced Salmonella loads in vital organs such as the liver, spleen, and cecum. This significant reduction suggests that M22 can effectively prevent pathogen translocation and subsequent systemic dissemination. Histopathological analysis further supported these findings, revealing that M22 treatment ameliorated hepatic inflammation, hepatocyte damage, and overall organ pathology induced by Salmonella infection. Similar protective effects were noted by Liu et al., who reported decreased organ pathology after treatment with probiotic-fermented milk [37]. Collectively, these results indicate that M22 provides systemic protection by inhibiting pathogen colonization, proliferation, and dissemination.

Inflammatory responses and oxidative stress are two pivotal pathological processes triggered during Salmonella infection, both of which play central roles in intestinal and systemic injury [38]. Our investigation into inflammatory cytokines and oxidative stress markers provided insights into the mechanisms underlying the protective effects of *P. acidilactici* M22. The significantly reduced levels of pro-inflammatory cytokines (TNF-α, IL-6, IL-1β) coupled with increased anti-inflammatory IL-10 in mice pretreated with M22 indicate effective modulation of the inflammatory response. Additionally, the observed reduction in oxidative stress markers (MDA) and enhanced antioxidant enzyme activities (SOD, GSH) demonstrate that M22 confers protection against Salmonella-induced oxidative damage. This dual modulation of inflammation and oxidative stress by probiotics has been well-documented in prior studies, further substantiating our findings [39,40].

Intestinal morphology serves as a critical indicator of mucosal integrity and barrier function, and structural alterations in the villi and crypts are closely associated with impaired nutrient absorption and host defense during enteric infections [41]. The morphological analysis of the jejunum further confirmed the protective effects of *P. acidilactici* M22 against *S.* Typhimurium-induced intestinal injury. Villus length and crypt depth are key indicators of absorptive function and epithelial turnover, and infection typically leads to villus atrophy, crypt hyperplasia, and a reduced villus-to-crypt (V/C) ratio, reflecting impaired nutrient absorption and disrupted barrier integrity [42]. In this study, *S.* Typhimurium infection caused significant villus shortening and crypt deepening, whereas M22 administration reversed these alterations by elongating villi, reducing crypt depth, and restoring the V/C ratio. Such improvements suggest that M22 alleviates excessive epithelial proliferation, promotes mucosal recovery, and maintains a favorable balance between absorptive and proliferative compartments. Consistent with findings from other lactic acid bacteria, these histological changes likely result from the combined effects of M22 on suppressing inflammation, modulating immune responses, and reinforcing barrier function [43]. Overall, the results provide strong evidence that *P. acidilactici* M22 protects jejunal morphology and contributes to intestinal health during pathogenic challenge.

Another significant protective mechanism observed was the reinforcement of intestinal barrier integrity. Disrupted expression of tight junction proteins, such as ZO-1 and Occludin, is a hallmark of compromised intestinal barriers, facilitating bacterial translocation [44]. Immunofluorescence results demonstrated that M22 administration restored tight junction protein expression, reinforcing the epithelial barrier against pathogenic breaches. This finding aligns with previous studies highlighting probiotics’ roles in enhancing tight junction integrity and maintaining intestinal homeostasis [21].

The intestinal microbial barrier is composed of a highly diverse community of microorganisms residing in the gut lumen [45]. In recent years, the gut microbiota has emerged as a central focus in health research [46]. By forming a symbiotic ecosystem, these microbes contribute to maintaining host homeostasis. Nevertheless, pathological disturbances of intestinal physiology can disrupt this balance and compromise barrier function [47]. Our 16S rRNA sequencing results demonstrated that *S.* Typhimurium infection caused marked dysbiosis of the cecal microbiota, characterized by decreased richness and diversity as indicated by reductions in the Chao1, Shannon, and Ace indices, along with a decline in the Simpson index reflecting reduced community evenness. Beta diversity analysis further revealed a distinct separation in microbial community structure between infected and C groups, confirming that *S.* Typhimurium substantially disrupts the gut microbial ecosystem. These findings align with existing literature demonstrating that enteric pathogens such as Salmonella trigger microbiota alterations conducive to pathogen survival and inflammatory responses, concomitant with a reduction in beneficial microbial populations [48,49,50]. Probiotic pretreatment with *P. acidilactici* M22 effectively mitigated these alterations, restoring alpha diversity indices and shifting beta diversity profiles toward those of healthy controls in a dose-dependent manner. Such recovery of microbial richness and evenness is critical for maintaining colonization resistance and metabolic stability in the gut. At the taxonomic level, *S.* Typhimurium challenge increased the relative abundance of *Verrucomicrobiota* and *potentially* harmful genera such as *Akkermansia*, *Helicobacter*, and *Desulfovibrio*, while depleting beneficial commensals including *Muribaculaceae*, *Lachnospiraceae*, and *Ligilactobacillus*. These changes are consistent with the pathogen-driven enrichment of mucin-degrading and pro-inflammatory taxa, coupled with loss of short-chain fatty acid (SCFA)-producing bacteria that contribute to barrier function and immune modulation. Importantly, *P. acidilactici* M22 pretreatment reversed many of these compositional changes, enriching SCFA-producing taxa (*Lachnospiraceae*, *Muribaculaceae*) and beneficial lactic acid bacteria (*Ligilactobacillus*) while reducing the abundance of inflammation-associated genera [51]. LEfSe analysis identified distinct microbial biomarkers for each group, with M22-supplemented mice exhibiting taxa associated with gut health, whereas the infected untreated group was enriched in taxa linked to dysbiosis and inflammation. Functional prediction using PICRUSt2 further suggested that *S.* Typhimurium infection impaired microbial pathways related to carbohydrate, amino acid, and energy metabolism, whereas M22 pretreatment partially or fully restored these functions, particularly in the H group. These results confirm that the protective effects of *P. acidilactici* M22 are at least partly mediated through restoration of gut microbial diversity, suppression of pathogenic or pro-inflammatory taxa, enrichment of beneficial commensals, and recovery of metabolic functional potential. This microbiota modulation likely synergizes with the observed anti-inflammatory, antioxidant, and barrier-protective effects, thereby contributing to comprehensive protection against *S.* Typhimurium infection [52].

While this study offers promising insights, it is not without its limitations. Although our findings are encouraging, several limitations warrant acknowledgment. First, the in vivo experiments were conducted in a mouse model rather than in *cats*, the natural host of the milk-derived strain. Although mice provide a well-established and controlled system for evaluating probiotic functions, the translational relevance to feline physiology may be limited. Therefore, further validation in *cats* or other target companion animals is warranted to confirm the applicability of these results. In addition, functional properties such as short-chain fatty acid production were not assessed in this study and should be explored in future investigations to provide a more comprehensive understanding of the probiotic potential of *P. acidilactici* M22.

## 5. Conclusions

In conclusion, our study highlights the preventive potential of *P. acidilactici* M22 against *S.* Typhimurium infection through multiple protective mechanisms, including reducing clinical symptoms, preventing systemic pathogen dissemination, modulating inflammatory responses and oxidative stress, restoring intestinal barrier integrity, and potentially modulating gut microbiota. These results validate the potential of strain M22 as a probiotic agent for the prevention of enteric infections and antibiotic reduction, with relevant applications in animal and public health settings—especially in mitigating zoonotic pathogen transmission.

## Data Availability

The original contributions presented in this study are included in the article. Further inquiries can be directed to the corresponding author.
